# New insights into the important roles of tumor cell-intrinsic PD-1

**DOI:** 10.7150/ijbs.60114

**Published:** 2021-06-16

**Authors:** Hongmei Zheng, Yue Ning, Yuting Zhan, Sile Liu, Qiuyuan Wen, Songqing Fan

**Affiliations:** Department of Pathology, The Second Xiangya Hospital, Central South University, Changsha, Hunan, 410011, China.

**Keywords:** PD-L1, tumor cell-intrinsic PD-1, immunotherapy

## Abstract

PD-1 (Programmed cell death protein-1) is mainly expressed in various immune cells, while its ligands PD-L1/PD-L2 (Programmed death ligand-1/Programmed death ligand-2) are mostly expressed in tumor cells. Generally, the binding of PD-L1/PD-L2 and PD-1 could lead to the tumor immune evasion. However, some recent studies showed that PD-1 could also be expressed in tumor cells and could activate mTOR (Mammalian Target of Rapamycin) or Hippo signaling pathway, therefore facilitating tumor proliferation independent of the immune system. While there was evidence that tumor cell-intrinsic PD-1 inhibited the activation of AKT and ERK1/2 pathways, thereby inhibiting tumor cell growth. Based on TCGA and CCLE database, we found that PD-1 was expressed in a variety of tumors and was associated with patient's prognosis. Besides, we found that PD-1 may be involved in many carcinogenic signaling pathway on the basis of PD-1 gene enrichment analysis of cancer tissues and cancer cells. Our understanding of the tumor cell-intrinsic PD-1 function is still limited. This review is aimed at elaborating the potential effects of tumor cell-intrinsic PD-1 on carcinogenesis, providing a novel insight into the effects of anti-PD-1/PD-L1 immunotherapy, and helping to open a major epoch of combination therapy.

## Introduction

PD-1 is a CD28 superfamily protein encoded by gene *PDCD1* on chromosome 2q37.3 1, 2. It is a cytomembrane protein composed of 268 amino acids with three domains, including transmembrane domain, immunoglobulin V-like domain and intracellular domain 3, 4. PD-L1 and PD-L2, two ligands of PD-1, pertain to B7 protein family, which are composed of 290 amino acids and 273 amino acids, respectively 2. It is generally believed that PD-L1/PD-L2 is mainly expressed in tumor and antigen-presenting cells, while PD-1 is often expressed in various immune cells, such as T lymphocytes, B lymphocytes and myeloid dendritic cells 5-7. In tumor microenvironment, the binding of PD-L1 and PD-1 negatively regulates T cell function and inhibits the production of cytokines, thus promoting tumor immune escape 8. Pathways PI3K/Akt and MAPK (Mitogen-activated protein kinase) as well as transcriptional factors STAT3, HIF-1 and NF-ĸB have been shown to facilitate the up-regulation of PD-L1 9. TGF-β1 (Transforming growth factor-beta 1) increases the PD-1 expression of antigen-specific T cell through Smad3 10. Anti-PD-L1/anti-PD-1 drugs can restore the effective immune response of immune cells to tumor, which have significantly improved the prognosis of patients with solid tumors. However, only a few patients have achieved long-term curative effect, and many patients eventually relapse due to drug resistance 11. Recent studies have shown that PD-1 could also be expressed in tumor cells 12, but its function remains to be further studied. Therefore, it is urgent and necessary to investigate the specific mechanisms of PD-1/PD-L1 axis, especially the function of tumor cell-intrinsic PD-1 independent of adaptive immunity. This review elaborates the potential effects of tumor cell-intrinsic PD-1 on carcinogenesis and its clinical significance.

## The structure and regulation of PD-1

PD-1 was first found in mouse apoptotic T cells in 1992. It is an inhibitory costimulatory molecule, also known as CD279, encoded by gene *PDCD1* on chromosome 2q37.3 1. PD-1 is a transmembrane protein, which consists of 268 amino acids with a relative molecular weight of 50-55 KDa. It consists of three parts: extracellular IgV-like domain, hydrophobic transmembrane domain and intracellular domain, including immunoreceptor tyrosine-based switch motif (ITSM) and immunoreceptor tyrosine-based inhibitory motif (ITIM) which are important structural sites for PD-1 to exert immunosuppressive effect 13, 14. PD-1 is mainly found on the surface of B cell, activated CD4^+^, CD8^+^ T cell and monocytes 5-7. The PD-1 expression of T cell surface could be induced by some cytokines such as IL-7, IL-15 and IL-21 15. miRNAs are a particular type of non-coding single-stranded RNA molecules encoded by endogenous genes and have about 22 nucleotides in length, which inhibit mRNA translation or directly degrade it by completely or partially pairing with complementary sequences on mRNA 3'UTR of the target gene 16. It has been found that the interaction of miR-4717 and PD-1 3'UTR single nucleotide polymorphism rs10204525 could down-regulate the expression of PD-1, affect the progress of chronic HBV infection, and change the immune status of the body at the same time 17. miR-28 and miR-138 have also been shown to inhibit the expression of PD-1 18, 19. Besides, TGF-β1 could enhance PD-1 expression on TILs (tumor infiltrating lymphocytes) through Smad3, thereby inducing tumor escape from host immune responses 10.

## The PD-1 signaling pathway

The complete immune systems could identify and eliminate tumor cells through immune examination, but tumors can adapt to and evade these defense mechanisms 20, 21. The combination of PD-L1 and PD-1 can phosphorylate the tyrosine in ITSM domain of PD-1, recruit SHP-1 (Src homology 2 domain-containing phosphatases-1) and SHP-2, and inhibit the activation of PI3K/Akt, which ultimately weakens the activation and proliferation of T lymphocytes, inhibits cytokine secretion and induces apoptosis of T lymphocytes 22, 23. PI3K/Akt signal is a significant downstream pathway of PD-1. PD-1 can induce PTEN phosphatase activity, thus inhibiting P13K/Akt pathway 24-26. PD-1 also prevents T cell from expressing PI3K-dependent anti-apoptotic protein Bcl-XL 27. Besides, PD-1 can block Ras/MEK/ERK pathway, thereby regulating cell cycle molecules and halting the proliferation of T cell 28. Interestingly, PD-1 inhibits PI3K/Akt pathway in a few minutes, while blocking Ras/MEK/ERK pathway takes several hours 29. In addition, PD-1 can hinder phosphorylation of ZAP70 and activation of PKC-θ, which are critical for T-cell activation, and prevent the development of effector T cells by promoting fatty acid oxidation and inhibiting glycolysis 30, 31. Also, PD-1/PD-L1 axis can activate and transmit anti-Fas apoptotic signal, affect the differentiation of dendritic cells and T lymphocytes, and activate the cytolytic effect of innate anti-CD8^+^ T lymphocytes 32. Furthermore, PD-1/PD-L1 axis can promote the development of iTreg (induced regulatory T) cell and increase Foxp3 expression on its surface, induce the differentiation of CD4^+^CD25^+^Foxp3^+^Treg and maintain its immunosuppressive function, thereby indirectly inhibiting T cells proliferation and promoting tumor immune evasion 33.

## Expression of PD-1 in cancer and its prognostic significance

It is widely believed that PD-1 is often expressed in activated CD4^+^ and CD8^+^ T cells, B cells, dendritic cells (DCs) and monocytes 5-7. In the tumor microenvironment, PD-1 is mainly expressed on the surface tumor-infiltrating lymphocytes. It has been demonstrated that PD-1 and AFAP1-AS1 (Actin filament-associated protein 1 antisense RNA 1) were co-expressed in the infiltrating lymphocytes of nasopharyngeal carcinoma tissues and patients with co-expression of AFAP1-AS1 and PD-1 had poor survival 34. Besides, it was confirmed by Li *et al*. that PD-1 of lymphocytes was an independent prognostic factor both for DFS and OS of colorectal cancer patients 35. Interestingly, PD-1 positivity of tumor-infiltrating lymphocytes was also an independent worse prognosis factor in patients with upper tract urothelial carcinoma 36.

**Figure 1:**
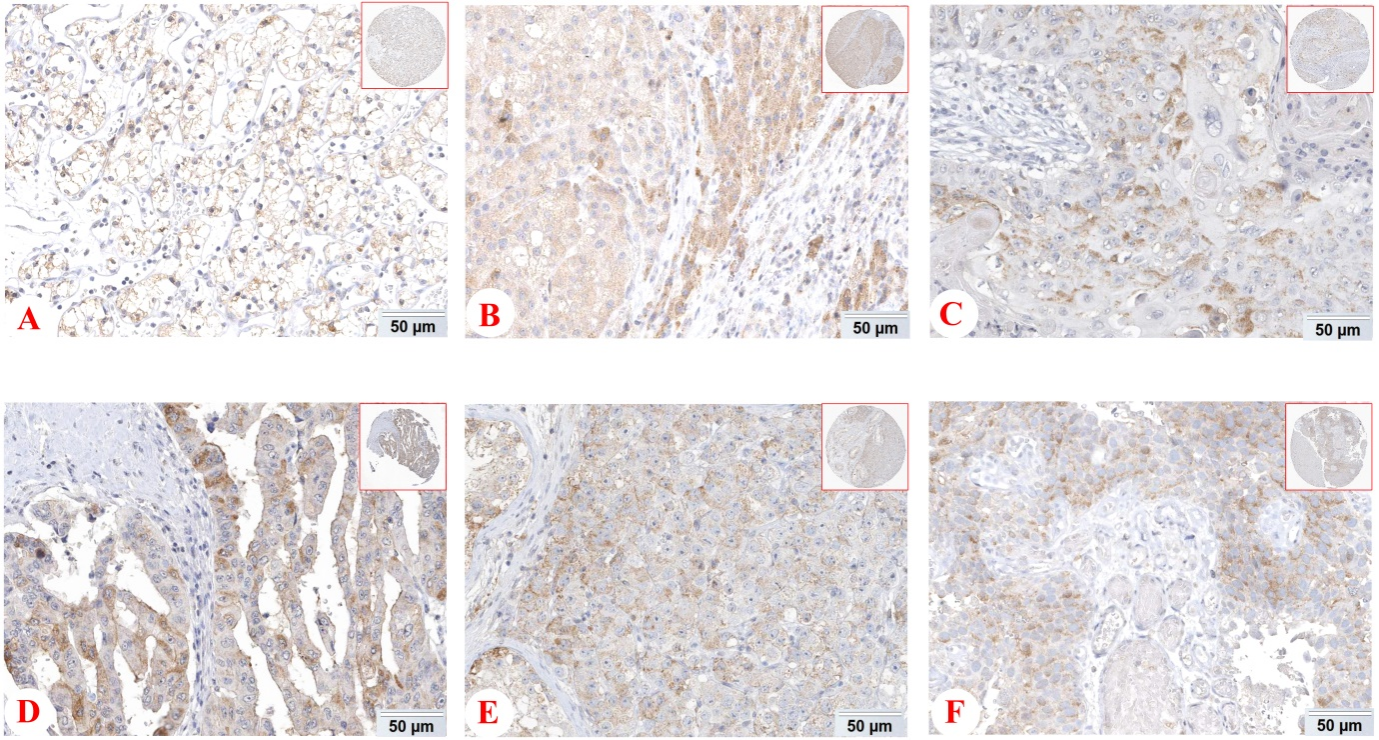
** Expression of PD-1 (*PDCD1*) protein in various cancer tissues from THE HUMAN PROTEIN ATLAS.** The expression of PD-1 protein in renal cancer (A), liver cancer (B), skin cancer (C), pancreatic cancer (D), testis cancer (E) and urothelial cancer (F).

However, some emerging studies have shown the expression and functions of PD-1 in tumor cells and tissues 12. The expession of PD-1 protein in different tissues from THE HUMAN PROTEIN ATLAS (https://www.proteinatlas.org/) was shown Figure 1. Li and his colleagues found that liver cancer cells and tissues could express PD-1, and hepatocellular carcinoma patients with low PD-1 expression had a higher survival rate and disease-free survival 37. In addition, there was also the expression of tumor cell-intrinsic PD-1 in NSCLC 38. Most notably, it has been demonstrated that PD-1 was also localized in cytoplasm and cell membrance of the pancreatic ductal adenocarcinoma cells and its expression was associated with OS 39. The main driver of tumor cell-intrinsic PD-1 expression remains unclear, but epigenetic alteration, gene amplification and posttranscriptional modification may be involved. In conclusion, PD-1 is expressed not only on tumor infiltrating lymphocytes, but also on tumor cells, which has shed new light on the precision treatment of tumors.

**Figure 2:**
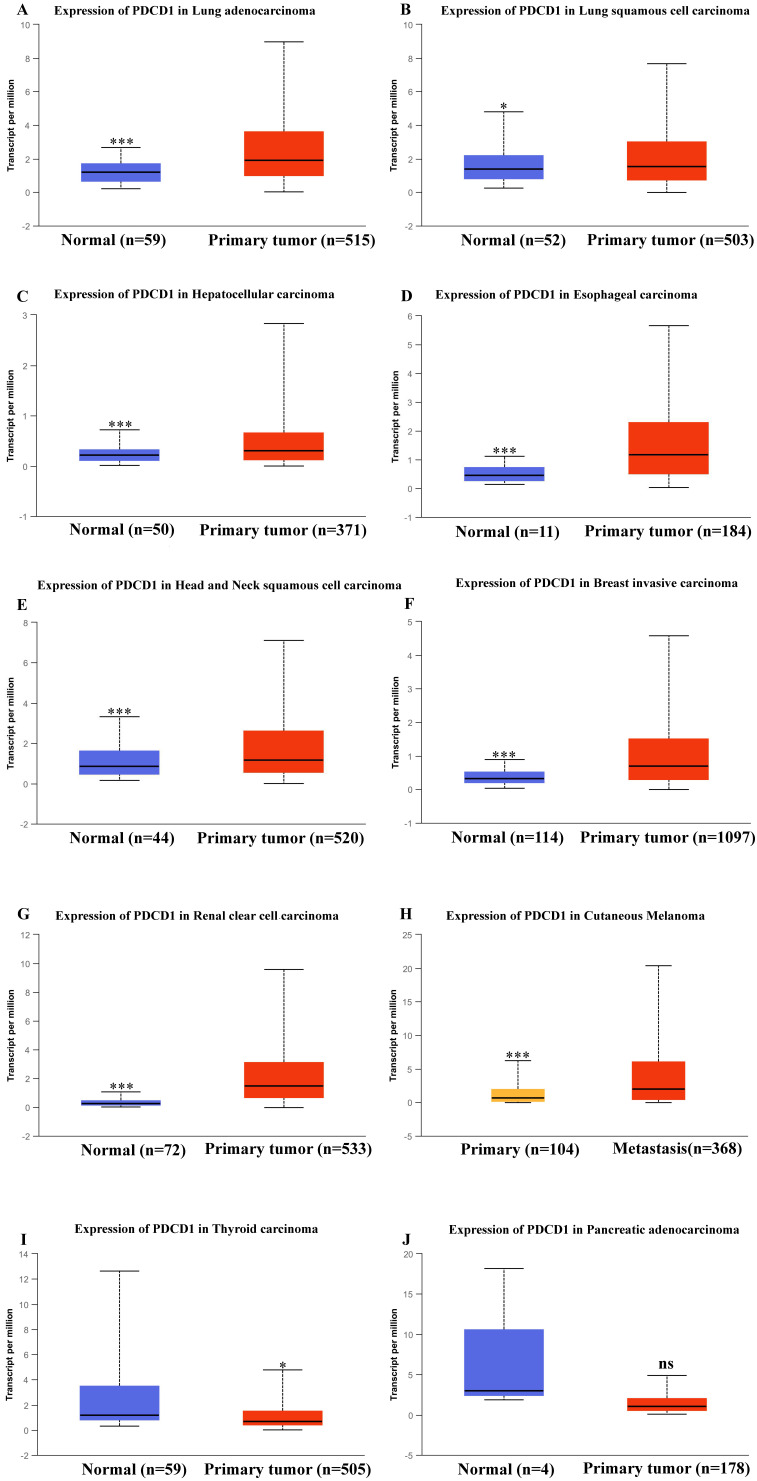
** mRNA expression of PD-1 (*PDCD1*) in various cancer tissues of TCGA database.** mRNA expression of *PDCD1* in lung adenocarcinoma (A), lung squamous cell carcinoma (B), hepatocellular carcinoma (C), esophageal carcinoma (D), head and neck squamous cell carcinoma (E), breast invasive carcinoma (F), renal clear cell carcinoma (G), cutaneous melanoma (H), thyroid carcinoma (I) and pancreatic adenocarcinoma (J).

**Figure 3:**
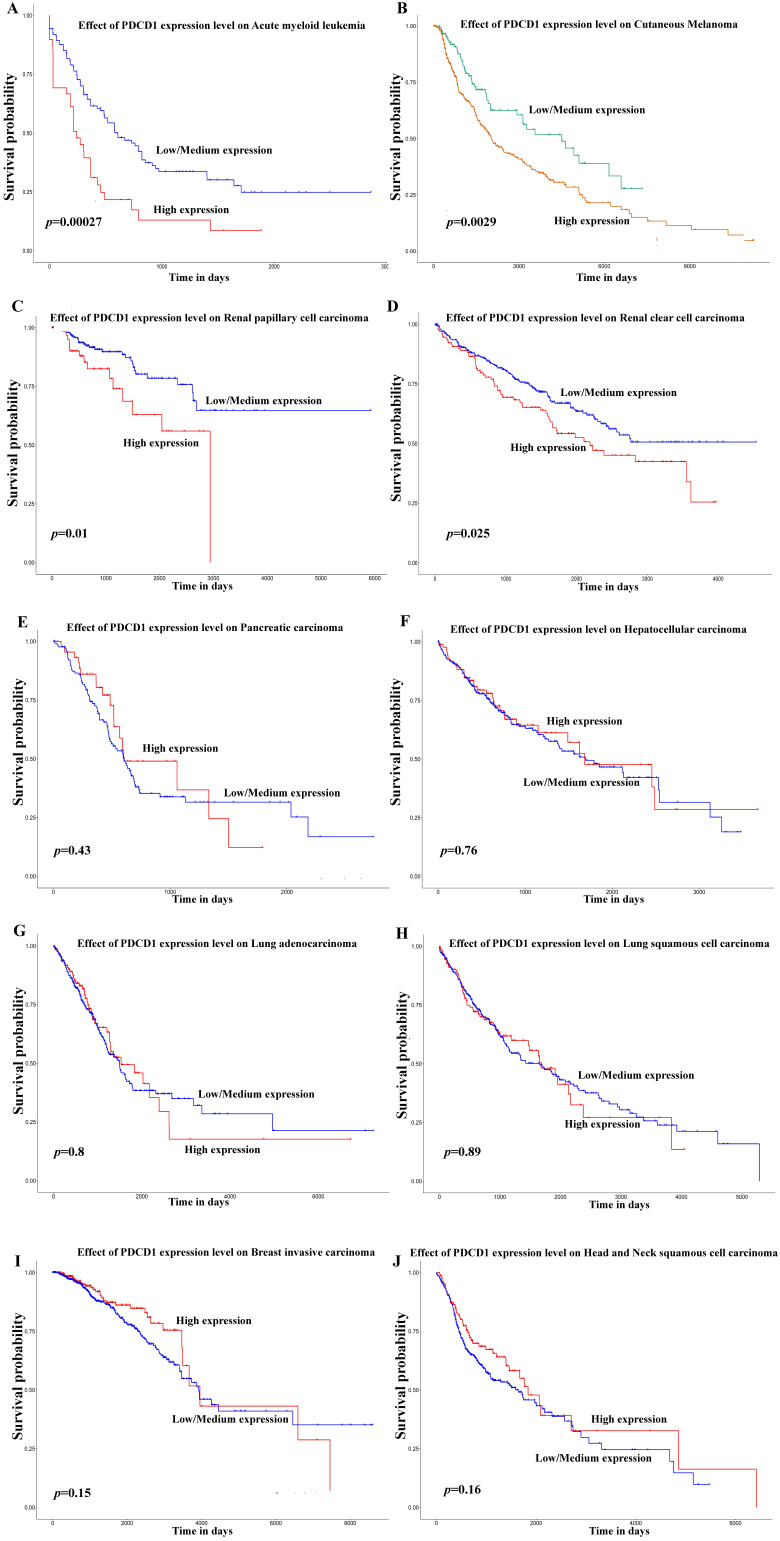
** Effect of PD-1 (*PDCD1*) on prognosis in different cancer patients of TCGA database.** Effect of *PDCD1* expression level on prognosis in patients with acute myeloid leukemia (A), cutaneous melanoma (B), renal papillary cell carcinoma (C), renal clear cell carcinoma (D), pancreatic carcinoma (E), hepatocellular carcinoma (F), lung adenocarcinoma (G), lung squamous cell carcinoma (H), breast invasive carcinoma (I), and head and neck squamous cell carcinoma (J).

Besides, in order to further expound the PD-1 mRNA level and its prognostic significance in various cancrs, we used UALCAN website (http://ualcan.path.uab.edu/) to analyse TCGA gene data. In our results, we found that mRNA expression of PD-1 (*PDCD1*) was evidently higher in lung adenocarcinoma/squamous cell carcinoma, hepatocellular carcinoma, esophageal carcinoma, head and neck squamous cell carcinoma, breast invasive carcinoma and renal clear cell carcinoma than that in normal control (Figure 2; all *P*<0.05). In addition, mRNA expression of PD-1 (*PDCD1*) was also elevated in metastasis cutaneous melanoma than that in primary cutaneous melanoma (*P*<0.0001). But, we also found that mRNA expression of PD-1 (*PDCD1*) was higher in normal control than that in thyroid carcinoma (*P*<0.05) but no different was found between pancreatic adenocarcinoma and its normal control (*P>*0.05). Furthermore, the results showed that high PD-1 (*PDCD1*) mRNA level patients had poor prognosis in acute myeloid leukemia, cutaneous melanoma and renal carcinoma (Figure 3; all *P*<0.05), but had no impact on pancreatic carcinoma, hepatocellular carcinoma, lung cancer and breast invasive carcinoma (all *P>*0.05).

## The roles of tumor cell-intrinsic PD-1

### Tumor cell-intrinsic PD-1 activates mTOR pathway

mTOR, a considerably conserved serine/threonine protein kinase, is one of the important substrates downstream of Akt 40, which exists in the form of mTORCl and mTORC2 complexes 41. mTORC1 consists of mTOR, raptor and mLST8 and regulates the biosynthesis of intracellular translation elements 42. mTOR signaling pathway is key to gene translation, which ultimately regulates cell growth and survival 43. Activated mTORC1 can phosphorylate its downstream 4E-BP1 and S6K1. Phosphorylated 4E-BP1 dissociates from eIF4E, makes elF4E free and promotes the transcription of proto-oncogenes such as c-myc and HIF-1α 44, 45. While phosphorylated S6K1 can continue to phosphorylate ribosomal protein S6, elF4B and other targets related to the translation of mRNA 46. In hepatocellular carcinoma, tumor cell-intrinsic PD-1 can active mTOR pathway, which binds to the downstream target molecules S6 and eIF4E and promotes their phosphorylation, thus promoting tumor progression independently from specific immunity 37. The findings put forward the possibility that PD-1 may be associated with cyclin D1 in tumor development by the regulation of p-eIF4E and suppress tumor necrosis factor-related apoptosis by regulating S6 37. Intriguingly, melanoma-expressed PD-1 activates mTOR signaling and promotes tumorigenesis by interacting with PD-L1 47.

### Tumor cell-intrinsic PD-1 activates Hippo pathway

Hippo signaling pathway regulates organ size through suppressing cell proliferation and increasing apoptosis and plays a significant role in development and homeostasis. Its main members include MST1/2, LATS1/2, SAV1, MOB1 and downstream effector molecules YAP and TAZ in mammals 48, 49. Dysregulation of this pathway is also related to the development of cancers. When Hippo pathway is activated, MST1/2 kinase binds to its ligand protein Sav and activates Sav. Then phosphorylated LATS1/2 kinase and MOB1 protein increase LATS/MOB1 complex and activate LATS1/2 kinase. Finally, activated LATS1/2 kinase phosphorylates and inactivates YAP and TAZ 50. On the contrary, dephosphorylated YAP/TAZ translocates into the nucleus, thereby induces the expression of target gene through binding to transcription factors, such as TEA domain family members 51, 52. In pancreatic cancer, tumor cell-intrinsic PD-1 interacts with MOB1 and decreases MOB1 phosphorylation, thereby suppressing the phosphorylation of LATS1 and inactivating LATS1, which inhibits YAP phosphorylation and accumulates YAP protein. And the accumulated YAP protein binds to TEDA and translocates into the nucleus, activating downstream genes of Hippo pathway, such as CYR61 and CTGF, which facilitates tumor proliferation independent of the immune system 39.

### Tumor cell-intrinsic PD-1 inhibits AKT and ERK1/2 pathways

p-AKT (phosphorylated protein kinase B) is a key molecule in activating PI3K/Akt signaling pathway that plays a crucial role in tumor development and progression 53. ERK1/2 (extracellular regulated protein kinase 1/2) is a member of MAPK (mitogen activated protein kinase) family. p-ERK1/2 (phosphorylated ERK1/2) can transfer to the nucleus, regulate the activity of transcription factors, and ultimately change the metabolism and function of cells and participate in forming malignant tumor cells 54-56. In lung cancer, tumor cell-intrinsic PD-1 is a tumor suppressor that inhibits the activation of AKT and ERK1/2 and thereby inhibits tumor cell growth 57. These evidences suggest that tumor cell-intrinsic PD-1 plays different roles in various tumors.

### PD-1 Gene enrichment analysis

#### Enrichment Analysis of Cancer Tissues

**Figure 4:**
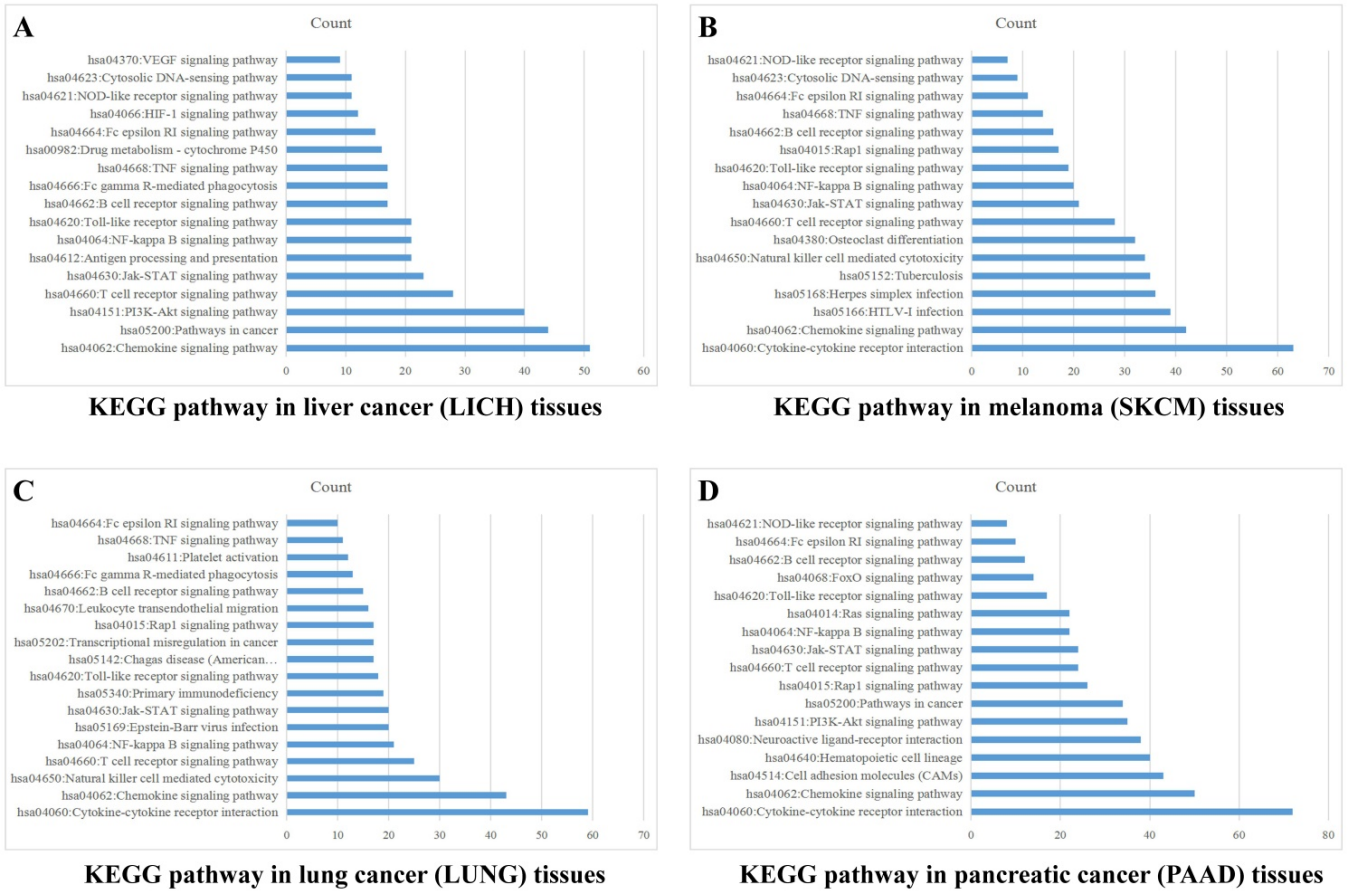
** PD-1 Gene enrichment analysis in different cancer tissues.** KEGG analysis of PD-1 in liver cancer tissues (A), melanoma tissues (B), lung cancer tissues (C) and pancreatic cancer tissues (D).

Sample data of liver cancer, melanoma, lung cancer and pancreatic cancer were downloaded from TCGA database, which were grouped into high and low expression on the basis of mean value of PD-1 (*PDCD1*) level. The differentially expressed genes were analyzed by analysis software DECenter (│logFC│>1, *P<*0.05). The GO and KEGG analysis about *PDCD1* gene were analyzed by online DAVID website (https://david.ncifcrf.gov/). The results showed that in liver cancer, there were 1175 differentially expressed genes, including 1067 up-regulation and 108 down-regulation. KEGG analysis found they were chiefly related to signaling pathways, such as PI3K-Akt, Chemokine, NF-kappa B, Jak-STAT, HIF-1, VEGF and TNF (Figure 4A). In melanoma, we found 779 differentially expressed genes, including 762 up-regulation and 17 down-regulation. KEGG found they were associated with signaling pathways, such as Chemokine, Jak-STAT, NF-kappa B, Toll-like receptor, Rap1, TNF and NOD-like receptor (Figure 4B). In lung cancer, there were 741 differentially expressed genes, including 52 up-regulation and 689 down-regulation. KEGG indicated they were related to signaling pathways, such as Chemokine, NF-kappa B, Jak-STAT, Rap1, TNF and Toll-like receptor (Figure 4C). In pancreatic cancer, there were 981 differentially expressed genes, including 23 up-regulation and 958 down-regulation. KEGG found they were related to signaling pathways, such as Chemokine, NF-kappa B, Jak-STAT, Toll-like receptor, PI3K-Akt and Ras (Figure 4D). GO analysis of PD-1 in these four cancers showed that differentially expressed genes were mainly involved in molecular functions related to immune response, signal transduction, cell adhesion, positive regulation of ERK1 and ERK2 cascade and GTPase activity.

#### Enrichment Analysis of Cancer Cells

**Figure 5:**
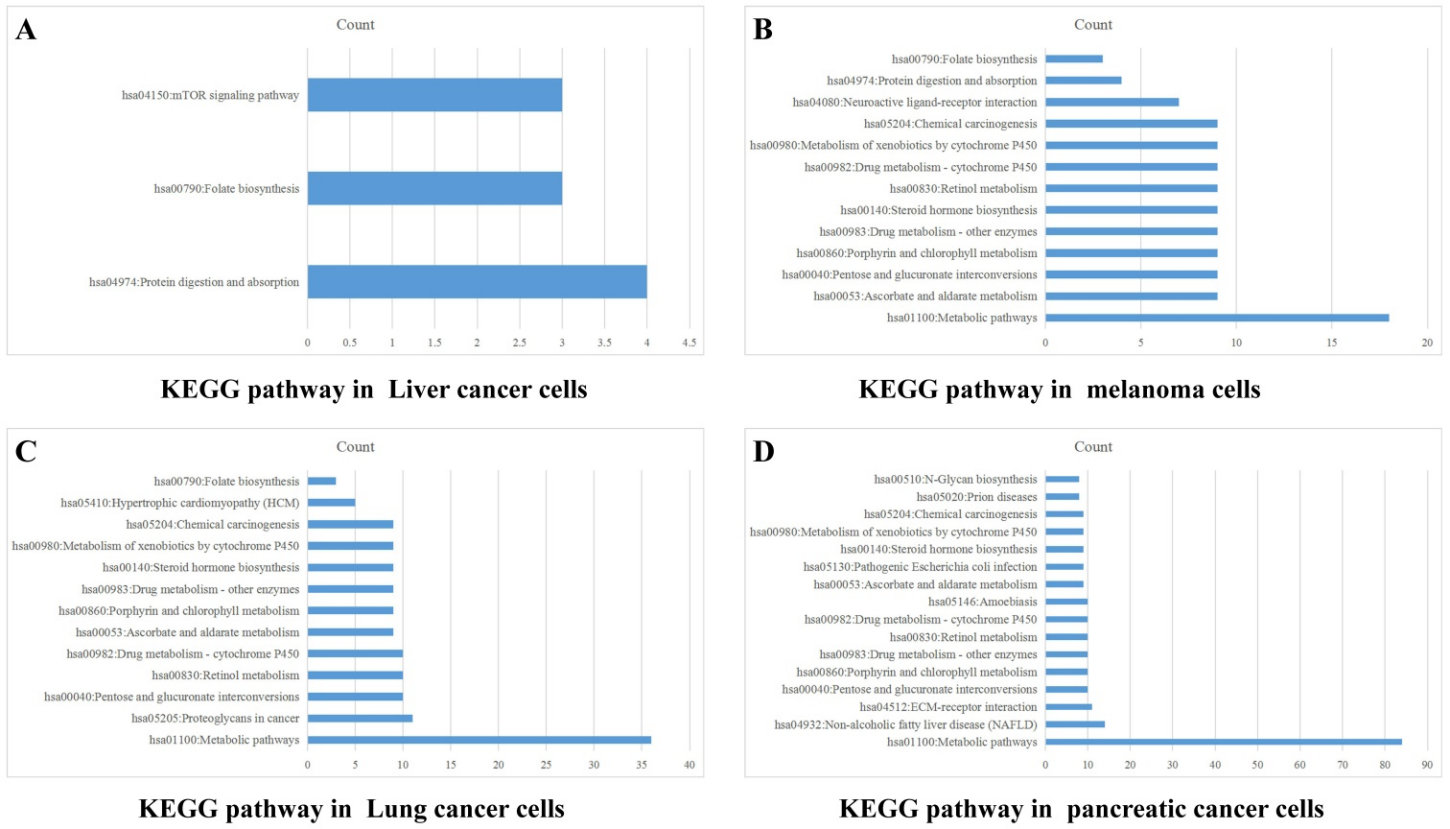
** PD-1 Gene enrichment analysis in different cancer cells.** KEGG analysis of PD-1 in liver cancer cells (A), melanoma cells (B), lung cancer cells (C) and pancreatic cancer cells (D).

Data from TCGA database may not precisely reflect the PD-1 (*PDCD1*) level of tumor cells, because there are a lot of infiltrating immune cells in tumor tissues. Therefore, we also analyzed PD-1 (*PDCD1*) expression in CCLE dataset to exclude the effect of PD-1 on immune cells. Data of PD-1 mRNA in lung cancer cells, liver cancer cells, melanoma cells and pancreatic cancer cells were grouped into high and low expression on the basis of mean value of PD-1. The differentially expressed genes were analyzed by software DECenter (|logFC|>0.1, *P*<0.05). The GO and KEGG analysis about PD-1 were analyzed by online DAVID website. The results showed that in liver cancer, there were 142 differentially expressed genes, including 137 up-regulation and 5 down-regulation. GO enrichment analysis showed they were relative to positive regulation of apoptotic process and G-protein coupled receptor signaling pathway. KEGG analysis found they were chiefly related to signaling pathways, such as mTOR, folate biosynthesis and protein digestion and absorption (Figure 5A). In melanoma, we found 165 differentially expressed genes, all of which were up-regulated genes. GO enrichment analysis indicated these genes were relative to metabolic process, intracellular signal transduction and retinoic acid metabolic process. KEGG analysis found they were connected with signal pathways, like drug metabolism-cytochrome P450, metabolic pathways and chemical carcinogenesis (Figure 5B). In lung cancer, there were 469 differentially expressed genes, including 419 up-regulation and 50 down-regulation. GO enrichment analysis indicated these genes were relative to intracellular signal transduction, positive regulation of transcription from RNA polymerase II promoter, angiogenesis and integrin-mediated signaling pathway. KEGG analysis indicated they were connected with retinol metabolism, metabolic pathways and proteoglycans in cancer (Figure 5C). In pancreatic cancer, there were 1129 differentially expressed genes, including 689 up-regulation and 440 down-regulation. GO enrichment analysis showed that they were relative to apoptotic process, response to drug, cell-cell adhesion, protein folding and metabolic process. KEGG analysis indicated they were connected with metabolic pathways, ECM-receptor interaction, retinol metabolism and chemical carcinogenesis (Figure 5D).

## Combination therapy based on PD-1 inhibitors

Currently, cancer immunotherapy is changing the clinical treatment of various cancers. PD-1 antibodies, such as, Nivolumab (Opdivo) and cemiplimab (Libtayo) and PD-L1 antibodies, like duravulumab and Atezolizumab, have been approved to be used in clinical application 58. Anti-PD-L1/PD-1 antibodies could relieve immunosuppression of T cells by binding with PD-1/PD-L1, thereby recovering T cell ability to kill tumor cells and normalizing the immune system 59, 60. However, only a proportion of patients could be beneficial from this immunotherapy with low response rate. More and more researches have shown that the combination of anti-PD-L1/PD-1 antibodies with other therapies may achieve better clinical results 58. CTLA-4 is different from PD-1 in inhibiting the activation of T cell 61. Greater clinical benefits can be obtained when combined with CTLA-4 and PD-1 in the treatment of metastatic melanoma and lung cancer 62. Besides, the combination of anti-PD-L1/PD-1 blockade with chemoradiotherapy has also shown promising results, especially in NSCLC patients, who have achieved higher overall efficacy and survival rate in the combined treatment 63. In addition to the tumor itself, gastrointestinal microbiome may also affect the response to immunotherapy. Some remarkable studies have reported that good gut bacteria were necessary for patient's response to anti-PD-1 therapy and thereby the combination with anti-microbiome modulation may be beneficial for immunotherapy 64, 65. Because PD-1 can also be expressed on tumor cells and activate some carcinogenic signaling pathway, many combination therapies based on this are also under development. For example, researchers find that in pancreatic ductal adenocarcinoma (PDAC), tumor cell-intrinsic PD-1 can facilitate tumor growth through hippo pathway and PD-1 checkpoint blockade combined with hippo pathway inhibitors can enhance its antitumor effect 39. Results from recent evidences demonstrate that tumor cell-intrinsic PD-1 can activate mTOR signal in liver cancer and combination therapy targeting mTOR pathways and PD-1 may have a better clinical efficacy 37. So far, there are many clinical studies about combination therapy based on PD-1 inhibitor Nivolumab, which is shown in Table 1.

Taken together, further clarifying the combination anti-PD-L1/PD-1 with other therapies will have synergistic effects and improve the clinical results of multiple cancer patients.

## Conclusion

For a long time, it has been widely believed that PD-1 is only expressed on immune cells and PD-L1 is mainly on tumor cells. With more and more in-depth researches, PD-1 on tumor cells has been revealed. Recent studies indicated PD-1 was found in liver cancer, lung cancer, melanoma and pancreatic cancer cells. Also, researchers found that tumor cell-intrinsic PD-1 may activate mTOR or Hippo signaling pathway, therefore facilitating tumor proliferation independent from immune system. However, tumor cell-intrinsic PD-1 can also inhibit the activation of AKT and ERK1/2 pathways, thereby inhibiting tumor cell growth. We used TCGA and CCLE database for enrichment analysis and found that PD-1 may be involved in many carcinogenic signaling pathway. Although there are increasing studies aimed at elucidating the roles of PD-1 and its clinical significance in tumor cells, our understanding about it is still limited. In the future, further researches on tumor cell-intrinsic PD-1 might offer a novel insight into the effects of anti-PD-L1/PD-1 immunotherapy, and help to open a major era of combination therapy.

## Figures and Tables

**Table 1:** Clinical trials of Nivolumab-based combination therapy

Combination drug	Clinical trial ID	Phase	Study title
Nivolumab+Azacitidine	NCT04128020	Phase 1	Azacitidine Plus Nivolumab Following Reduced-intensity Allogeneic PBSC Transplantation for Patients With AML and High-risk Myelodysplasia
Nivolumab+Avadomide (CC-122)	NCT03834623	Phase 2	Avadomide (CC-122) in Combination With Nivolumab in Advanced Melanoma
Nivolumab+Testosterone cypionate	NCT03554317	Phase 2	COMbination of Bipolar Androgen Therapy and Nivolumab (COMBAT-CRPC)
Nivolumab+Tivozanib	NCT03136627	Phase 1/2	Phase 1/2 Study of Tivozanib in Combination With Nivolumab in Subjects With RCC
Nivolumab+Ipilimumab	NCT03651271	Phase 2	Treatment With Nivolumab and Ipilimumab or Nivolumab Alone according to the Percentage of Tumoral CD8 Cells in Advanced Metastatic Cancer
Nivolumab+Ramucirumab	NCT03502746	Phase 2	Phase II Nivolumab and Ramucirumab for Patients With Previously-Treated Mesothelioma
Nivolumab+Ipilimumab	NCT03789110	Phase 2	NIMBUS: Nivolumab Plus Ipilimumab in Metastatic Hypermutated HER2-negative Breast Cancer
Nivolumab+ Regorafenib	NCT03712943	Phase 1	Regorafenib and Nivolumab in Mismatch Repair (MMR) Refractory Colorectal Cancer
Nivolumab+Cabozantinib	NCT04197310	Phase 2	Cabozantinib and Nivolumab for Carcinoid Tumors
Nivolumab+Anlotinib Hydrochloride	NCT04503967	Phase 2	Efficacy and Safety of Anlotinib Hydrochloride Combined With Nivolumab in the Treatment of Gastric and Esophageal Cancer (OASIS)
Nivolumab+Pemetrexed	NCT04107103	Phase 2	Nivolumab Plus Pemetrexed for Head and Neck Squamous Cell Carcinoma (NivoPlus)
Nivolumab+HF10	NCT03259425	Phase 2	Neoadjuvant Trial of Nivolumab in Combination With HF10 Oncolytic Viral Therapy in Resectable Stage IIIB, IIIC, IVM1a Melanoma
Nivolumab+HBI-8000	NCT02718066	Phase 1/2	Study of HBI-8000 With Nivolumab in Melanoma, Renal Cell Carcinoma and Non-Small Cell Lung Cancer
Nivolumab+Copanlisib	NCT03884998	Phase 1	Copanlisib and Nivolumab in Treating Participants With Richter's Transformation or Transformed Indolent Non-Hodgkin's Lymphoma
Nivolumab+APX005M	NCT03123783	Phase 1/2	CD40 Agonistic Antibody APX005M in Combination With Nivolumab
Nivolumab+ACY-241	NCT02635061	Phase 1	Selective HDAC6 Inhibitor ACY 241 in Combination With Nivolumab in Patients With Unresectable Non-Small Cell Lung Cancer

## Abbreviations

PD-1: programmed cell death protein-1
PD-L1: programmed death ligand-1
PD-L2: programmed death ligand-2
mTOR: Mammalian Target of Rapamycin
TCGA: The Cancer Genome Atlas
MAPK: mitogen-activated protein kinase
STAT3: Signal Transducer and Activator of Transcription 3
HIF-1: hypoxia inducible factor-1
NF-ĸB: nuclear factor kappa-B
TGF-β1: transforming growth factor-beta 1
ITSM: immunoreceptor tyrosine-based switch motif
ITIM: immunoreceptor tyrosine-based inhibitory motif
SHP-1: Src homology 2 domain-containing phosphatases-1
iTreg: induced regulatory T
DCs: dendritic cells
NKT: natural killer T cells
AFAP1-AS1: actin filament-associated protein 1 antisense RNA 1
NPC: nasopharyngeal carcinoma
OS: overall survival
DFS: disease free survival
NSCLC: non-small cell lung cancer
mLST8: mammalian ortholog of LST8
4E-BP1: eukaryotic initiation factor 4E-binding protein 1
S6K1: p70 ribosomal protein S6 kinase B1
eIF4E: eukaryotic initiation factor 4E
elF4B: eukaryotic initiation factor 4B
MST1/2: mammalian STE20-like protein kinase 1/2
LATS1/2: large tumor suppressor 1/2
SAV1: Salvador family WW domain-containing protein 1
MOB1: MOB kinase activator 1
YAP: Yes-associated protein
TAZ: transcriptional coactivator with a PDZ-binding domain
TEADs: TEA domain family members
CYR61: cysteine-rich angiogenic inducer 61
CTGF: connective tissue growth factor
CCLE: Cancer Cell Line Encyclopedia
CTLA-4: Cytotoxic T lymphocyte associated antigen-4
PDAC: Pancreatic ductal adenocarcinoma

## References

[1] Ishida Y, Agata Y, Shibahara K (1992). Induced expression of PD-1, a novel member of the immunoglobulin gene superfamily, upon programmed cell death. EMBO J.

[2] Ok CY, Young KH (2017). Targeting the programmed death-1 pathway in lymphoid neoplasms. Cancer Treat Rev.

[3] Daeron M, Jaeger S, Du Pasquier L (2008). Immunoreceptor tyrosine-based inhibition motifs: a quest in the past and future. Immunol Rev.

[4] El FA, Voisin T, Rouyer-Fessard C (2009). Discovery of a functional immunoreceptor tyrosine-based switch motif in a 7-transmembrane-spanning receptor: role in the orexin receptor OX1R-driven apoptosis. FASEB J.

[5] Azuma T, Yao S, Zhu G (2008). B7-H1 is a ubiquitous antiapoptotic receptor on cancer cells. Blood.

[6] Fourcade J, Sun Z, Benallaoua M (2010). Upregulation of Tim-3 and PD-1 expression is associated with tumor antigen-specific CD8+ T cell dysfunction in melanoma patients. J Exp Med.

[7] Yao S, Chen L (2014). PD-1 as an immune modulatory receptor. Cancer J.

[8] Kim JW, Eder JP (2014). Prospects for targeting PD-1 and PD-L1 in various tumor types. Oncology (Williston Park).

[9] Chen J, Jiang CC, Jin L (2016). Regulation of PD-L1: a novel role of pro-survival signalling in cancer. Ann Oncol.

[10] Park BV, Freeman ZT, Ghasemzadeh A (2016). TGFbeta1-Mediated SMAD3 Enhances PD-1 Expression on Antigen-Specific T Cells in Cancer. Cancer Discov.

[11] Meyers DE, Bryan PM, Banerji S (2018). Targeting the PD-1/PD-L1 axis for the treatment of non-small-cell lung cancer. Curr Oncol.

[12] Yao H, Wang H, Li C (2018). Cancer Cell-Intrinsic PD-1 and Implications in Combinatorial Immunotherapy. Front Immunol.

[13] Okazaki T, Honjo T (2007). PD-1 and PD-1 ligands: from discovery to clinical application. Int Immunol.

[14] Sidorenko SP, Clark EA (2003). The dual-function CD150 receptor subfamily: the viral attraction. Nat Immunol.

[15] Kinter AL, Godbout EJ, McNally JP (2008). The common gamma-chain cytokines IL-2, IL-7, IL-15, and IL-21 induce the expression of programmed death-1 and its ligands. J Immunol.

[16] Ambros V (2004). The functions of animal microRNAs. Nature.

[17] Zhang G, Li N, Li Z (2015). microRNA-4717 differentially interacts with its polymorphic target in the PD1 3' untranslated region: A mechanism for regulating PD-1 expression and function in HBV-associated liver diseases. Oncotarget.

[18] Li Q, Johnston N, Zheng X (2016). miR-28 modulates exhaustive differentiation of T cells through silencing programmed cell death-1 and regulating cytokine secretion. Oncotarget.

[19] Wei J, Nduom EK, Kong LY (2016). MiR-138 exerts anti-glioma efficacy by targeting immune checkpoints. Neuro Oncol.

[20] Disis ML (2010). Immune regulation of cancer. J Clin Oncol.

[21] Dolan DE, Gupta S (2014). PD-1 pathway inhibitors: changing the landscape of cancer immunotherapy. Cancer Control.

[22] Carter L, Fouser LA, Jussif J (2002). PD-1:PD-L inhibitory pathway affects both CD4(+) and CD8(+) T cells and is overcome by IL-2. Eur J Immunol.

[23] Cheng X, Veverka V, Radhakrishnan A (2013). Structure and interactions of the human programmed cell death 1 receptor. J Biol Chem.

[24] Patsoukis N, Li L, Sari D (2013). PD-1 increases PTEN phosphatase activity while decreasing PTEN protein stability by inhibiting casein kinase 2. Mol Cell Biol.

[25] Torres J, Pulido R (2001). The tumor suppressor PTEN is phosphorylated by the protein kinase CK2 at its C terminus. Implications for PTEN stability to proteasome-mediated degradation. J Biol Chem.

[26] Vazquez F, Ramaswamy S, Nakamura N (2000). Phosphorylation of the PTEN tail regulates protein stability and function. Mol Cell Biol.

[27] Parry RV, Chemnitz JM, Frauwirth KA (2005). CTLA-4 and PD-1 receptors inhibit T-cell activation by distinct mechanisms. Mol Cell Biol.

[28] Patsoukis N, Brown J, Petkova V (2012). Selective effects of PD-1 on Akt and Ras pathways regulate molecular components of the cell cycle and inhibit T cell proliferation. Sci Signal.

[29] Saunders PA, Hendrycks VR, Lidinsky WA (2005). PD-L2:PD-1 involvement in T cell proliferation, cytokine production, and integrin-mediated adhesion. Eur J Immunol.

[30] Patsoukis N, Bardhan K, Chatterjee P (2015). PD-1 alters T-cell metabolic reprogramming by inhibiting glycolysis and promoting lipolysis and fatty acid oxidation. Nat Commun.

[31] Sheppard KA, Fitz LJ, Lee JM (2004). PD-1 inhibits T-cell receptor induced phosphorylation of the ZAP70/CD3zeta signalosome and downstream signaling to PKCtheta. FEBS Lett.

[32] Chen L, Han X (2015). Anti-PD-1/PD-L1 therapy of human cancer: past, present, and future. J Clin Invest.

[33] Park HJ, Kusnadi A, Lee EJ (2012). Tumor-infiltrating regulatory T cells delineated by upregulation of PD-1 and inhibitory receptors. Cell Immunol.

[34] Tang Y, He Y, Shi L (2017). Co-expression of AFAP1-AS1 and PD-1 predicts poor prognosis in nasopharyngeal carcinoma. Oncotarget.

[35] Li Y, Liang L, Dai W (2016). Prognostic impact of programed cell death-1 (PD-1) and PD-ligand 1 (PD-L1) expression in cancer cells and tumor infiltrating lymphocytes in colorectal cancer. Mol Cancer.

[36] Krabbe LM, Heitplatz B, Preuss S (2017). Prognostic Value of PD-1 and PD-L1 Expression in Patients with High Grade Upper Tract Urothelial Carcinoma. J Urol.

[37] Li H, Li X, Liu S (2017). Programmed cell death-1 (PD-1) checkpoint blockade in combination with a mammalian target of rapamycin inhibitor restrains hepatocellular carcinoma growth induced by hepatoma cell-intrinsic PD-1. Hepatology.

[38] Du S, McCall N, Park K (2018). Blockade of Tumor-Expressed PD-1 promotes lung cancer growth. Oncoimmunology.

[39] Pu N, Gao S, Yin H (2019). Cell-intrinsic PD-1 promotes proliferation in pancreatic cancer by targeting CYR61/CTGF via the hippo pathway. Cancer Lett.

[40] Hare SH, Harvey AJ (2017). mTOR function and therapeutic targeting in breast cancer. Am J Cancer Res.

[41] Zou Z, Chen J, Yang J (2016). Targeted Inhibition of Rictor/mTORC2 in Cancer Treatment: A New Era after Rapamycin. Curr Cancer Drug Targets.

[42] Carneiro BA, Kaplan JB, Altman JK (2015). Targeting mTOR signaling pathways and related negative feedback loops for the treatment of acute myeloid leukemia. Cancer Biol Ther.

[43] Laplante M, Sabatini DM (2012). mTOR signaling in growth control and disease. Cell.

[44] Probst HC, McCoy K, Okazaki T (2005). Resting dendritic cells induce peripheral CD8+ T cell tolerance through PD-1 and CTLA-4. Nat Immunol.

[45] Wan X, Helman LJ (2007). The biology behind mTOR inhibition in sarcoma. Oncologist.

[46] Holz MK, Ballif BA, Gygi SP (2005). mTOR and S6K1 mediate assembly of the translation preinitiation complex through dynamic protein interchange and ordered phosphorylation events. Cell.

[47] Kleffel S, Posch C, Barthel SR (2015). Melanoma Cell-Intrinsic PD-1 Receptor Functions Promote Tumor Growth. Cell.

[48] Liu H, Du S, Lei T (2018). Multifaceted regulation and functions of YAP/TAZ in tumors (Review). Oncol Rep.

[49] Kriz V, Korinek V (2018). Wnt, RSPO and Hippo Signalling in the Intestine and Intestinal Stem Cells. Genes (Basel).

[50] Hansen CG, Ng YL, Lam WL (2015). The Hippo pathway effectors YAP and TAZ promote cell growth by modulating amino acid signaling to mTORC1. Cell Res.

[51] Piccolo S, Dupont S, Cordenonsi M (2014). The biology of YAP/TAZ: hippo signaling and beyond. Physiol Rev.

[52] Hansen CG, Moroishi T, Guan KL (2015). YAP and TAZ: a nexus for Hippo signaling and beyond. Trends Cell Biol.

[53] Zheng H, Zhan Y, Zhang Y (2019). Elevated expression of G3BP1 associates with YB1 and p-AKT and predicts poor prognosis in nonsmall cell lung cancer patients after surgical resection. Cancer Med.

[54] Collier JB, Schnellmann RG (2020). Extracellular signal-regulated kinase 1/2 regulates NAD metabolism during acute kidney injury through microRNA-34a-mediated NAMPT expression. Cell Mol Life Sci.

[55] Roskoski RJ (2019). Targeting ERK1/2 protein-serine/threonine kinases in human cancers. Pharmacol Res.

[56] Urosevic J, Blasco MT, Llorente A (2020). ERK1/2 Signaling Induces Upregulation of ANGPT2 and CXCR4 to Mediate Liver Metastasis in Colon Cancer. Cancer Res.

[57] Wang X, Yang X, Zhang C (2020). Tumor cell-intrinsic PD-1 receptor is a tumor suppressor and mediates resistance to PD-1 blockade therapy. Proc Natl Acad Sci U S A.

[58] Jiang Y, Chen M, Nie H (2019). PD-1 and PD-L1 in cancer immunotherapy: clinical implications and future considerations. Hum Vaccin Immunother.

[59] Page DB, Postow MA, Callahan MK (2014). Immune modulation in cancer with antibodies. Annu Rev Med.

[60] Sanmamed MF, Chen L (2018). A Paradigm Shift in Cancer Immunotherapy: From Enhancement to Normalization. Cell.

[61] Wei SC, Levine JH, Cogdill AP (2017). Distinct Cellular Mechanisms Underlie Anti-CTLA-4 and Anti-PD-1 Checkpoint Blockade. Cell.

[62] Ribas A, Wolchok JD (2018). Cancer immunotherapy using checkpoint blockade. Science.

[63] Mathew M, Enzler T, Shu CA (2018). Combining chemotherapy with PD-1 blockade in NSCLC. Pharmacol Ther.

[64] Gopalakrishnan V, Spencer CN, Nezi L (2018). Gut microbiome modulates response to anti-PD-1 immunotherapy in melanoma patients. Science.

[65] Matson V, Fessler J, Bao R (2018). The commensal microbiome is associated with anti-PD-1 efficacy in metastatic melanoma patients. Science.

